# Genetic variants of the promoter of the heme oxygenase-1 gene and their influence on cardiovascular disease (The Ludwigshafen Risk and Cardiovascular Health Study)

**DOI:** 10.1186/1471-2350-10-36

**Published:** 2009-04-23

**Authors:** Nicola Lüblinghoff, Karl Winkler, Bernhard R Winkelmann, Ursula Seelhorst, Britta Wellnitz, Bernhard O Boehm, Winfried März, Michael M Hoffmann

**Affiliations:** 1 Department of Otorhinolaryngology – Head and Neck Surgery, University Medical Center Freiburg, Freiburg, Germany; 2 Division of Clinical Chemistry, Department of Medicine, University Medical Center Freiburg, Freiburg, Germany; 3 Cardiology Group, Frankfurt-Sachsenhausen, Germany; 4 LURIC Study nonprofit LLC, Freiburg, Germany; 5 Division of Endocrinology and Diabetes, Department of Medicine, Ulm University, Ulm, Germany; 6 Synlab Center of Laboratory Diagnostics, Heidelberg, Germany; 7 Institute of Public Health, Medical Faculty of Mannheim, University of Heidelberg, Heidelberg, Germany

## Abstract

**Background:**

Heme oxygenase-1 is an inducible cytoprotective enzyme which handles oxidative stress by generating anti-oxidant bilirubin and vasodilating carbon monoxide. A (GT)_n _dinucleotide repeat and a -413A>T single nucleotide polymorphism have been reported in the promoter region of *HMOX1 *to both influence the occurrence of coronary artery disease and myocardial infarction. We sought to validate these observations in persons scheduled for coronary angiography.

**Methods:**

We included 3219 subjects in the current analysis, 2526 with CAD including a subgroup of CAD and MI (n = 1339) and 693 controls. Coronary status was determined by coronary angiography. Risk factors and biochemical parameters (bilirubin, iron, LDL-C, HDL-C, and triglycerides) were determined by standard procedures. The dinucleotide repeat was analysed by PCR and subsequent sizing by capillary electrophoresis, the -413A>T polymorphism by PCR and RFLP.

**Results:**

In the LURIC study the allele frequency for the -413A>T polymorphism is A = 0,589 and T = 0,411. The (GT)n repeats spread between 14 and 39 repeats with 22 (19.9%) and 29 (47.1%) as the two most common alleles. We found neither an association of the genotypes or allelic frequencies with any of the biochemical parameters nor with CAD or previous MI.

**Conclusion:**

Although an association of these polymorphisms with the appearance of CAD and MI have been published before, our results strongly argue against a relevant role of the (GT)_n _repeat or the -413A>T SNP in the *HMOX1 *promoter in CAD or MI.

## Background

Low-grade inflammation and oxidation are important mechanisms involved in the complex pathological process of atherogenesis [1]. Several cardiovascular risk factors, such as smoking, type 2 diabetes, hypertension, and dyslipidemia, increase the production of reactive oxygen species (ROS) in the arterial wall. In response to oxidative stress the expression of endogenous stress proteins including heme oxygenase (HO) is increased. HO is a rate-limiting enzyme in the degradation of heme to biliverdin releasing equimolar quantities of free iron and carbon monoxide, a potent anti-apoptotic and proliferation promoting compound [2]. The reduction of biliverdin by biliverdin reductase leads to the generation of bilirubin, a potent endogenous antioxidant [3,4]. Recently, it could be shown that bilirubin has an antiproliferative effect on vascular smooth muscle cells, too [5].

In humans two isoforms of heme oxygenase, HO-1 and HO-2, have been described. HO-1 is the inducible isoform, which is up-regulated by various exogenous stimuli, such as oxidative stress, hypoxia, or its own substrate heme [6,7]. The enzyme is expressed in atherosclerotic lesions, vascular smooth muscle cells, endothelial cells, and several other tissues [8]. There are several lines of evidence that HO-1 has protective properties in the cardiovascular system. HO-1 provides protection against cellular proliferation and lesion formation after arterial injury [9]. In rats vascular remodelling following balloon injury was beneficially influenced after induction of HO-1 [10]. In addition, thrombus formation was suppressed by vascular HO-1 [11] and HO-1 inhibited vascular smooth muscle cell proliferation in vitro [12]. Overexpression of HO-1 in endothelial cells has not only improved the ability of these cells to resist oxidative stress but also enhances cell growth [13]. Thus, the up regulation of HO-1 in atherosclerotic lesions might be a physiological reaction to prevent atherosclerotic progression [14].

Two different polymorphisms in the *HMOX1 *promoter, a (GT) dinucleotide repeat and the -413A>T (rs2071746) single nucleotide polymorphism (SNP) are thought to be involved in the pathogenesis of cardiovascular disease by modulating the expression of HO-1 [15-24].

We investigated whether these variants are associated with angiographic coronary artery disease (CAD), myocardial infarction (MI), or mortality in a large cohort of individuals who had previously undergone coronary angiography.

## Methods

### Study design and participants

The **Lu**dwigshafen **Ri**sk and **C**ardiovascular Health (LURIC) study includes consecutive white patients hospitalized for coronary angiography between July 1997 and January 2000. The study was approved by the ethics review committee at the "Landesärztekammer Rheinland-Pfalz" (Mainz, Germany). Written informed consent was obtained from each of the participants.

A detailed description of LURIC has been published [25]. Diabetes mellitus was diagnosed according to the criteria of the American Diabetes Association (ADA) [26]. Further, individuals with a history of diabetes or treatment with oral antidiabetics or insulin were considered diabetic. CAD has been defined angiographically using the maximum luminal narrowing estimated by visual analysis. Clinically relevant CAD was defined as the presence of a visible luminal narrowing (≥ 20% stenosis) in at least one of 15 coronary segments according to a classification of the American Heart Association [27]. We also provisionally defined CAD as the presence of at least one stenosis of 50 percent or more, again classifying individuals with stenosis less than 20 percent as controls. Previous MI was diagnosed if there was a conclusive positive history of myocardial infarction or if patients presented ST elevation. Hypertension was diagnosed if the systolic and/or diastolic blood pressure exceeded 140 and/or 90 mm Hg or if there was a history of hypertension, evident through the use of antihypertensive drugs. Individuals with either HDL cholesterol (HDL-C) <40 mg/dl or LDL cholesterol (LDL-C) >160 mg/dl or triglycerides >200 mg/dl were considered dyslipidemic.

We included 3219 patients in this study; all of them underwent coronary angiography. To rule out racial differences as a confounding factor the LURIC study was designed to recruit probands with German ancestry only. We did not include family members of the patients for this study.

Information on vital status was obtained from local person registries. No patients were lost to follow-up. Amongst the subjects included in this examination, 752 deaths (23.4%) had occurred during a median observation time of 7.97 years. Cardiovascular death (n = 473) included the following categories: sudden death, fatal myocardial infarction, death due to congestive heart failure, death immediately following intervention to treat CAD, fatal stroke and other causes of death due to CAD. All clinical assessments were made blinded to the knowledge of HMOX1 genotype.

### Laboratory Procedures

Fasting blood samples were obtained by venipuncture in the early morning. Levels of cholesterol, triglycerides, bilirubin, iron, transferrin, and ferritin were determined with common laboratory procedures [25]. LDL-C and HDL-C were measured after separating lipoproteins with a combined ultracentrifugation-precipitation method.

### Analysis of the HO-1 promoter variants

Genomic DNA was prepared from EDTA-anticoagulated peripheral blood by using a standard salting-out procedure. Genotypes of the -413A>T polymorphism were determined using polymerase chain reaction with a mismatched 5' primer to obtain an allele specific restriction site for *Hind*III in the amplicon (sense 5'-gtt cct gat gtt gcc cac ca**a**gct-3'; reverse 5'-tct gag aag ctg cag gct ctg-3'). In case of the A genotype a fragment of 163 bp and in case of the T genotype two fragments of 140 bp + 23 bp were obtained. To validate the method three samples of each genotype were sequenced; as internal control 184 DNA samples, blinded distributed within the samples, were genotyped twice. To confirm genotype assignment, the restriction fragments were analyzed independently by two scientists on two separate occasions. The results were scored blinded as to case-control status.

The microsatellite polymorphism was amplified according to the protocol described by Kaneda et al. [18] and analyzed on a MegaBASE500 sequencer. As controls two cloned alleles (22 & 30 repeats) were run in parallel. The respective sizes of the (GT)_n _repeats were each calculated using Genetic Profiler software (Amersham).

### Statistics

Clinical and anthropometric characteristics were compared between CAD patients and controls by analysis of variance (ANOVA) or logistic regression. Covariable adjustments were carried out as indicated. Associations between categorical variables were examined by chi-square testing or logistic regression analysis. The frequencies of the *HMOX1 *alleles and genotypes in CAD+ and CAD- as well as in MI+ and MI- subjects were obtained by direct count and the deviation from Hardy-Weinberg equilibrium was examined by the chi-square test. In models assuming a co-dominant (additive) effect of the A-allele of the *HMOX1 *gene, the genotypes AA, AT and TT were coded as 0, 1, and 2, respectively. Genotypes were either treated as interval-scaled or categorical variables, the AA genotype being considered as the reference category in the latter case. When assuming a dominant effect, genotype TT was coded as 0, and AT and AA combined as 1, whereas assuming a recessive effect, genotypes TT and AT were coded as 0, and AA as 1. In a similar way the repeat classes (cut off S<25, L= 25, or S<27, L= 27, respectively) and both haplotypes (22-T, 29-A) were analyzed. To examine the relationship of the genotypes with total mortality and mortality from cardiovascular causes, we calculated hazard ratios and 95 percent confidence intervals (95% CI) using the Cox proportional hazards model. The criterion for statistical significance was p < 0.05. The SPSS statistical package (SPSS Inc., Chicago, IL, USA, version 15.0 for Windows XP) was used for all statistical analyses.

## Results

### Study participants – Baseline Characteristics

We included 3219 subjects in the current analysis. 693 were control subjects without CAD, 2526 were diagnosed CAD-positive by angiography and from these 1339 had suffered and survived a MI in the past. Compared to the control group, patients with CAD and with a former MI were significantly older, current or past smoker and type 2 diabetes were more prevalent (Table 1). Systemic Hypertension was only significantly higher in the CAD group compared to the control group, but not in the CAD and MI group. No significant differences were found for BMI.

**Table 1:** Baseline characteristics and risk factors in patients with angiographically proven CAD, and previous MI as compared to controls without CAD.

		**Controls**	**CAD**	**p**	**CAD and MI**	**p**
	Male	(n = 360)	(n = 1891)		(n = 1060)	
	Female	(n = 333)	(n = 635)		(n = 279)	

Age (years) means ± SD	Male	55 ± 12	63 ± 10		63 ± 10	
	Female	62 ± 10	66 ± 10	<0.001^1^	65 ± 10	<0.001^1^
Body mass index (kg/m^2^) means ± SD	Male	27 ± 4	28 ± 4		27 ± 4	
	Female	27 ± 5	27 ± 5	n.s.^2^	27 ± 5	n.s.^2^
Smoker (former and current)	Male	239 (66%)	1487 (78%)		867 (82%)	
	Female	100 (30%)	253 (39%)	<0.001^4^	145 (51%)	<0.001^4^
Diabetes mellitus	Male	63 (17%)	653 (34%)		374 (35%)	
	Female	66 (20%)	253 (39%)	<0.001^4^	120 (42%)	<0.001^4^
Systemic Hypertension	Male	212 (58%)	1399 (74%)		736 (69%)	
	Female	228 (69%)	519 (81%)	0.002^4^	220(77%)	n.s.^4^

Fasting blood glucose (mmol/L) means ± SD	Male	5,89 ± 1,67	6,39 ± 1,89		6,34 ± 1,83	
	Female	5,78 ± 1,34	6,6 ± 2,44	<0.001^2^	6,67 ± 2,5	<0.001^2^
LDL-C(mmol/L) means ± SD	Male	3,02 ± 0,8	2,92 ± 0,85		2,84 ± 0,83	
	Female	3,2 ± 0,8	3,2 ± 1,01	0.002^3^	3,05 ± 1,01	n.s.^3^
HDL-C (mmol/L) means ± SD	Male	1,03 ± 0,28	0,93 ± 0,23		0,9 ± 0,23	
	Female	1,19 ± 0,31	1,09 ± 0,28	<0.001^3^	1,03 ± 0,28	<0.001^3^
Bilirubin (μmol/L) means ± SD	Male	13,17 ± 7,53	11,46 ± 6,33		10.95 ± 5.82	
	Female	9.58 ± 4,62	8,89 ± 6,16	<0.001^2^	8,38 ± 4,10	<0.001^2^
Iron (μmol/L)	Male	19,52 ± 8.06	16,66 ± 6,98		16,12 ± 6,98	
	Female	17,19 ± 6,09	14,69 ± 5,91	<0.001^2^	13,97 ± 5,91	<0.001^2^
Triglycerides (mmol/L) median (25^th ^and 75^th ^percentile)	Male	1,64 (1,16–2,4)	1,69 (1,26–2,27)		1,7 (1,26–2,25)	
	Female	1,44 (1,04–2,01)	1,73 (1,3–2,34)	<0.012^3^	1,75 (1,31–2,46)	n.s.^3^

^1^analysis of variance adjusted for gender only

^2^analysis of variance adjusted for age and gender

^3^analysis of variance additionally adjusted for use of lipid-lowering drugs

^4^logistic regression, adjusted for age and gender

CAD and CAD plus MI patients had higher fasting glucose, lower HDL-C, lower bilirubin serum levels and lower iron serum concentrations. After adjustment for use of lipid lowering drugs CAD patients had – compared to the control group – higher LDL-C (p < 0.002) and higher triglycerides (p < 0.012). No significant difference was found for the two last mentioned parameters for the CAD plus MI group (Table 1).

With regard to the tested biochemical parameters there were no significant differences between the CAD and the CAD plus MI patients.

### -413A>T polymorphism

The overall allelic distribution in the LURIC study is A = 0,589 and T = 0,411. The genotypes were in Hardy-Weinberg equilibrium in both the CAD and the control group. There was no significant difference in the frequency of genotypes between CAD patients and controls (Table 2). Using logistic regression analysis to examine a possible association between the *HMOX1 *polymorphism and CAD we found no evidence for this association, regardless of whether or not we adjusted for conventional cardiovascular risk factors like age, gender, smoking, type 2 diabetes, dyslipidemia and hypertension (Table 3). Very similar results were obtained when we compared the -413A>T genotypes in patients with previous MI versus controls or CAD patients without MI (data not shown).

**Table 2:** Prevalence of HMOX1 genotypes and haplotypes in CAD patients and controls.

**HMOX1 genotype**	**Controls**		**CAD**		***P*^1^**
	**N**	**%**	**N**	**%**	

**A>T rs2071746**					

AA	246	35.5	893	35.4	

AT	341	49.2	1181	46.8	

TT	106	15.3	452	17.9	0.245

A allele	833	60.1	2967	58.7	

T allele	553	39.9	2085	41.3	0.358

**VNTR S <25, L ≥ 25 repeats**					

SS	66	9.5	286	11.3	

SL	302	43.6	1070	42.4	

LL	325	46.9	1170	46.3	0.399

S allele	434	31.3	1642	32.5	

L allele	952	68.7	3410	67.5	0.402

**VNTR S <27, L ≥ 27 repeats**					

SS	93	13.4	370	14.6	

SL	328	47.3	1157	45.8	

LL	272	39.2	999	39.5	0.651

S allele	514	37.1	1897	37.5	

L allele	872	62.9	3155	62.5	0.752

**Haplotype 22-T**					

0	455	65.7	1631	64.6	

1	209	30.2	774	30.6	

2	29	4.2	121	4.8	0.752

other haplotypes	1119	80.7	4036	79.9	

22 – T	267	19.3	1016	20.1	0.484

**Haplotype 29 – A**					

0	185	26.7	718	28.4	

1	356	51.4	1243	49.2	

2	152	21.9	565	22.4	0.566

other haplotypes	726	52.4	2679	53.0	

29 – A	660	47.6	2373	47.0	0.669

^1 ^Chi-squaredtest

**Table 3:** Odds ratios (OR) for angiographic CAD according to HMOX1 genotypes and haplotypes.

	**Model 1** **OR (95% CI)**	***P***	**Model 2** **OR (95% CI)**	***P***	**Model 3** **OR (95% CI)**	***P***
**A>T rs2071746**						
AA	1.0^reference^		1.0^reference^		1.0^reference^	
AT	0.95 (0.79–1.15)	0.619	0.92 (0.76–1.12)	0.398	1.03 (0.82–1.30)	0.788
TT	1.18 (0.91–1.52)	0.215	1.16 (0.89–1.52)	0.282	1.27 (0.93–1.73)	0.142
AA vs AT vs TT^2^	1.06 (0.94–1.19)	0.363	1.04 (0.92–1.19)	0.506	1.11 (0.95–1.28)	0.190
AA vs AT or TT^1^	1.21 (0.96–1.52)	0.110	1.22 (0.95–1.55)	0.115	1.24 (0.94–1.65)	0.135
AA or AT vs TT^1^	1.01 (0.84–1.20)	0.963	0.98 (0.81–1.17)	0.789	1.09 (0.88–1.35)	0.455

**VNTR S<25, L ≥ 25**						
SS	1.0^reference^		1.0^reference^		1.0^reference^	
SL^1^	0.82 (0.61–1.10)	0.183	0.76 (0.56–1.05)	0.086	0.70 (0.49–1.019	0.056
LL^1^	0.83 (0.62–1.12)	0.217	0.80 (0.59–1.09)	0.153	0.71 (0.49–1.02)	0.063
SS vs SL vs LL^2^	0.95 (0.84–1.08)	0.407	0.95 (0.83–1.09)	0.413	0.90 (0.77–1.05)	0.187
SS vs SL or LL^1^	0.82 (0.62–1.09)	0.180	0.78 (0.58–1.05)	0.100	0.71 (0.50–1.00)	0.049
SS or SL vs LL^1^	0.98 (0.83–1.16)	0.787	0.99 (0.83–1.19)	0.945	0.95 (0.77–1.16)	0.592

^1^Genotypes treated as categorical variables and compared to the reference category.

^2^Genotypes treated as interval-scaled variables.

Model 1: unadjusted.

Model 2: adjusted for age, and sex.

Model 3: in addition adjusted for clinical status at presentation (no CAD, stable CAD, unstable CAD, NSTEMI, STEMI), body mass index, metabolic syndrome/diabetes mellitus, hypertension, smoking, LDL cholesterol, HDL cholesterol, triglycerides (log transformed), SAA, use of betablockers, use of lipid lowering drugs.

### (GT)_n _repeat

In the study group the (GT)_n _repeats spread between 14 and 39 repeats with 22 and 29 as the two most common alleles, which represents a shift of minus one repeat in comparison to published data. Although we used cloned and sequenced alleles as controls we cannot exclude that this is a methodical mistake.

According to the classification of Funk et al. [28], we chose a cut-off at 25 and in a second model according to Kaneda et al. [18] a cut-off at 27 repeats: class S (small) < 25 or 27 repeats and class L (large) ≥ 25 or 27 repeats, respectively. Similar to the results presented for the -413A>T polymorphism we found no significant differences in the distribution of the repeats (Table 2). In a logistic regression analysis no association could be found with CAD (Table 3) or MI (data not shown), irrespective of the chosen cut-off.

Chen et al. [17] described an association of the L allele (≥ 32 repeats) with CAD in type 2 diabetic patients. In a subgroup analysis of type 2 diabetic patients (n = 1035) we could not confirm this observation, the L alleles (≥ 32 repeats) were evenly distributed between cases and controls (allele frequency 5.2% versus 5.5%, p = 0.619).

### Haplotypes

The small repeats, e.g. 22 – the most frequent ones in the class S alleles – are associated with the T allele, while the large repeats, e.g. 29, with the exception of very large repeats (34–38), are associated with the A allele (pairwise LD r^2 ^= 0,63). Analysis of the two most common haplotypes, 22-T (19.9%) and 29-A (47.1%), did not generate new information with regard to cardiovascular disease; there was still no association with CAD or MI (data not shown).

We obtained similar results by using a stenosis of at least 50% in the coronary segments as the criterion for CAD in all of the above mentioned analysis (data not shown).

### HMOX1 variants, all cause and cardiovascular mortality

Amongst the 3219 subjects included in this examination, 752 deaths (23.4%) had occurred during a median observation time of 7.97 years, from which 473 (14.7%) died from cardiovascular causes. Regardless whether or not we adjusted for age and gender, there was neither an association of the genetic markers of *HMOX1 *with mortality from all causes nor with cardiovascular mortality (Table 4).

**Table 4:** Hazard ratios (HR) for total and cardiac mortality according to HMOX1 genotypes and haplotypes.

	**HR (95% CI)** Total*	***P***	**HR (95% CI)** Cardiac*	***P***
**A>T rs2071746**				
AA	1.0^reference^		1.0^reference^	
AT^1^	0.99 (0.84–1.16)	0.886	1.06 (0.87–1.29)	0.569
TT^1^	0.96 (0.78–1.19)	0.727	0.95 (0.73–1.26)	0.734
AA vs AT or TT^1^	0.97 (0.80–1.18)	0.746	0.92 (0.72–1.18)	0.519
AA or AT vs TT^1^	0.98 (0.85–1.14)	0.818	1.03 (0.86–1.25)	0.739

**VNTR S<25, L ≥ 25**				
SS	1.0^reference^		1.0^reference^	
SL^1^	1.08 (0.84–1.40)	0.543	1.12 (0.81–1.54)	0.501
LL^1^	1.08 (0.84–1.39)	0.569	1.02 (0.74–1.40)	0.922
SS vs SL or LL^1^	1.08 (0.85–1.38)	0.536	1.06 (0.79–1.44)	0.688
SS or SL vs LL^1^	1.01 (0.87–1.16)	0.910	0.93 (0.77–1.11)	0.423

^1^Genotypes treated as categorical variables and compared to the reference category.

*adjusted for age, and sex.

### HMOX1 variants and biochemical parameters

Products of the enzymatic action of HO-1 are iron and biliverdin, which in turn is converted to bilirubin. If promoter variants influence the systemic expression of HO-1 it might be possible to detect an association between *HMOX1 *genotypes and serum levels. However, we were not able to find any significant association between *HMOX1 *genotypes and serum levels of bilirubin (e.g. -413A>T and bilirubin (μmol/L): AA 10.78 ± 6.50, AT 11.12 ± 6.67, and TT 10.95 ± 5.99, means ± standard deviations) or iron, even after adjustment for age, gender, or other cardiovascular risk factors.

Previously a correlation of lipid parameters and the (GT)_n _repeat has been published [29]. To avoid a confounding by lipid lowering drugs we analyzed only those patients (n = 1648) not receiving lipid lowering therapy. We found a significant increase of HDL-C (mmol/L) only for homozygote carriers of the 29-A haplotype (0 – 1.02 ± 0.28, 1 – 1.03 ± 0.29, 2 – 1.08 ± 0.05, means ± standard deviations), which was robust against adjustment for age and gender (p = 0.006), but no association with LDL-C or triglycerides.

## Discussion

The presented data show in a prospective case-control study of more than 3000 participants that neither the (GT)_n _dinucleotide repeat nor the -413A>T polymorphism in the *HMOX1 *promoter are associated with angiographic CAD, MI or survival rate in Caucasians undergoing coronary angiography.

The functional relevance of both variants in the *HMOX1 *promoter is still under debate. Yamada et al. described a higher response of reporter gene constructs with short (GT) repeats to H_2_0_2 _exposure in A549 and Hep3B cell [30]. Similar results were obtained by analyzing HO-1 mRNA and activity in lymphoblastoid cell lines from subjects homozygous for small or long repeats [31] or by analyzing the HO-1 expression in mononuclear cells from patients who underwent coronary angiography [24]. In all studies the short repeats were associated with a higher expression rate. However, Ono et al. [21,32] showed by *in vitro *experiments with reporter gene constructs that the length of the (GT)_n _repeat did not influence the activity of the *HMOX1 *promoter in bovine aortic endothelial cells. They found that – instead of the (GT)_n _repeat – the responsible element was an A>T exchange at position -413, giving rise to a 4-fold increase of activity. Therefore further experiments are required to clear the functionality of these variants.

Irrespective of the unclear functional role there are several reports suggesting a role of the (GT)_n _repeat in the *HMOX1 *promoter in cardiovascular disease. Asian patients with a lower number of repeats were less susceptible to pulmonary emphysema and coronary artery disease [17,18,30]. Further, a lower number of repeats was associated with a lower restenosis rate after balloon dilatation in the femoral artery [15] suggesting a protective effect on restenosis. In support of this hypothesis, long repeats have been related to in-stent-restenosis after percutanous coronary intervention [20,22]. However, data of Wijpkema et al. [33], who followed a group of about 3.000 patients for 10 months after successful percutanous coronary intervention, did not confirm the previous observations. Recently, the results of Wijpkema at el. have been confirmed by Tiroch et al, who studied 1357 patients 6 months after coronary stenting [34]. Like these two groups and in contrast to several smaller studies, we have not been able to confirm any influence of the (GT)_n _microsatellite in the promoter of the *HMOX1 *gene on the risk of CAD, a history of MI, and total or cardiovascular mortality.

It is not unusual, that association studies of genetic polymorphisms produce divergent results, especially if small numbers of cases and controls are examined. In many instances positive associations seen in small studies have been disproven in subsequent studies of larger cohorts. Both, our CAD and control group were substantially larger than the groups investigated in the other reports. The only report with a comparable number of participants is the study of Wijpkema et al. and their results are in line with ours [33]. In addition, the reliability of the former studies is challenged by the creation of three subclasses [17,30,31] or different cut-offs like S<23 [17], S<25 [15,23], S<26 [20,24], S<27 [18], and S<29 [22] without any rational explanation for these classifications. Especially in small cohorts the cut-off can substantially affect risk estimation. To avoid this problem we examined two common cut-offs (S<25 and S<27), with the minor allele representing 32.2% or 37.5%, respectively, and found no association with cardiovascular disease, irrespective of the chosen model. Therefore, we are confident that previous observations have overestimated the effect of the (GT)_n _repeat.

Concerning the -413A>T SNP only one study has been published before [21] showing a decreased risk of ischemic heart disease for homozygous carriers of the A allele in Japanese individuals. First, we found a switch in the allele distribution in Caucasians towards A as the major allele (A = 0,589 versus A = 0,442 in Japanese), confirming data of the HapMap consortium for Caucasians, and second we could not confirm any of the described associations. This is not unusual because it is well known that there is a significant genetic diversity between Caucasians and Asians. Furthermore the number of cases in the Japanese study was very low (MI = 393, angina pectoris = 204) [21].

On product of the enzymatic action of HO-1 is biliverdin, which in turn is converted to bilirubin. Biliverdin and bilirubin are both antioxidants, and higher serum levels of bilirubin are inversely related to the risk of CAD [14]. Actually, a similar relationship can be found in our study, the control group showing significantly higher serum levels of bilirubin (Table 1). In contrast to the hypothesis that genetic variants of *HMOX1 *associated with a higher expression would increase bilirubin serum levels we did not see any association between *HMOX1 *genotypes and bilirubin concentration. We can not exclude that other genes involved in bilirubin metabolism superpose the effect of different *HMOX1 *genotypes. One candidate gene is *UGT1A1*, coding for bilirubin uridine diphosphate-glucuronosyltransferase. This enzyme is responsible for the glucuronidation of bilirubin and thus enhances its elimination. Recently, a promoter variation (UGT1A1*28) has been associated with higher bilirubin serum levels and lower risk for coronary heart disease in the Framingham Heart Study [35]. The metabolism of iron is even more complicated and therefore it is not surprising that we did not see any correlation between *HMOX1 *genotypes and serum iron levels.

A methodological aspect of the present study that deserves discussion, like in many case-control association studies, is the appropriateness of the control group. Because angiography is relatively insensitive for early lesions that do not impact on the vascular lumen, early lesions undetectable by angiography could not be ruled out in controls, similar to coronary microvascular dysfunction. Nonetheless, coronary angiography remains a gold standard for the diagnosis of CAD, and for the definition of the coronary artery status, this may however be regarded as a strength of the study because of "controls" were also studied by angiography. This is crucial because the prevalence of clinically asymptomatic coronary atherosclerosis has been reported to be high at or above the age of 50 years [36]. Hence, angiography based recruitment of controls rules out that individuals with significant, yet clinically unapparent CAD are inadvertently allocated in the control group. Surprisingly, our control group appears well representative since the major cardiovascular risk factors occur at a similar frequency as in the general population [37].

## Conclusion

In conclusion, the regulation of the *HMOX1 *gene may be determined, at least in part, by genetics, and reduced ability to induce HO-1 expression may be involved in the mechanism of coronary atherosclerosis. Nevertheless, neither the (GT)_n _dinucleotide repeat nor the -413A>T polymorphism of *HMOX1 *gene are reliable genetic markers for cardiovascular disease in Caucasians.

## Competing interests

The authors declare that they have no competing interests.

## Authors' contributions

NL carried out the microsatellite analysis and contributed to the analysis and interpretation of the data as well as to the writing of the manuscript. KW participated in the design of the study and helped to perform the statistical analysis and to draft the manuscript. US and BW organised the follow-up within LURIC and contributed the survival data. BRW, BOB, and WM conceived of the LURIC study and participated in its design and coordination. MMH participated in the design of the study, carried out the SNP analysis, performed the statistical analysis and drafted the manuscript. All authors read and approved the final manuscript.

## Pre-publication history

The pre-publication history for this paper can be accessed here:


